# Beyond National Averages: Assessing the Applicability of a National Rapid Response Registry to a Mid-sized Urban Community Teaching Hospital

**DOI:** 10.7759/cureus.107969

**Published:** 2026-04-29

**Authors:** John Bajouka, Keyur Patel, Rana Afram, Jessica Lavoie, Avery Mendelson

**Affiliations:** 1 Internal Medicine, Henry Ford Health System, Southfield, USA

**Keywords:** mortality, patient-centered outcomes, patient safety, quality improvement, rapid response team

## Abstract

Rapid response teams (RRTs) manage deteriorating patients. National benchmarks, like the GWTG-R-MET (Get With The Guidelines-Resuscitation - Medical Emergency Team) registry, guide RRT practices, but their applicability to diverse settings like mid-sized community teaching hospitals is unclear. This study compares RRT characteristics at such a hospital to national registry data to assess applicability and identify local distinctions. We analyzed RRT activations (n = 912) at our institution (July 2022-March 2024) using rapid response (RR) sheets and electronic health record data, including demographics, triggers, disposition, mortality, and International Classification of Diseases, Tenth Edition (ICD-10) coding. Data were recoded to align with published GWTG-R-MET categories (n = 347,401) for direct comparison using chi-squared tests (p < 0.05, significant). Compared to the registry, our hospital had significantly more activations for non-Hispanic Black patients (75.9% vs. 19.7%, p < 0.001) and fewer for non-Hispanic White patients (17.8% vs. 67.2%, p < 0.001). Neurological triggers were higher locally (41.7% vs. 30.7%, p < 0.001), driven by altered mental status and stroke suspicion. Chest pain triggers were markedly higher (16.1% vs. 4.6%, p < 0.001), while respiratory triggers were significantly lower (12.7% vs. 38.0%, p < 0.001). More patients remained on their unit post-RRT (70.4% vs. 54.9%, p < 0.001), fewer transferred to ICU (21.5% vs. 30.0%, p < 0.001), and inpatient mortality was lower (5.8% vs. 14.3%, p < 0.001). National RRT registries provide a valuable framework for understanding RRT practices, but significant local variations in patient demographics, activation patterns, disposition, and outcomes exist. The observed variations highlight the necessity for each hospital to undertake a localized RRT needs assessment to inform tailored improvement strategies and education programs.

## Introduction

Concern over preventable morbidity and mortality in hospitalized patients grew following the identification of suboptimal care preceding intensive care unit (ICU) admissions in the late 1990s [1]. Since then, management of hospitalized patients has become increasingly complex with advances in interventions, increasing patient age, and increasing comorbidities [2]. These complexities contribute to adverse in-hospital events, including unplanned ICU admissions, cardiopulmonary arrest, and death [3]. Of note, studies have suggested that the majority of these mortalities may be preventable and are often preceded by signs of clinical deterioration, such as hypotension, tachycardia, tachypnea, decreased oxygen saturation, and altered mentation [4-6]. Recognizing this growing public health concern, the Institute for Healthcare Improvement launched the 100,000 Lives Campaign in 2004, promoting adoption of rapid response teams (RRTs) in US hospitals as a systemic solution to address preventable morbidity and mortality [7].

RRTs are specialized groups trained to assess deteriorating patients and intervene promptly to prevent further decline. While there is significant variation in the composition of these teams, they typically consist of critical care nurses, respiratory therapists, and physicians; in many institutions, RRTs are led by mid-level practitioners or trainees, often without senior physician supervision. In addition to varying composition, activation protocols vary widely as well, with the most common being clinical concerns, vital sign abnormalities, and early warning scores [8]. This institutional variability complicates outcome tracking and guideline development, hindering efforts to standardize RRT efficacy.

The “Get With the Guidelines-Resuscitation” (GWTG-R), a national registry launched by the American Heart Association (AHA) in 2005, aims to establish national benchmarks, monitor trends in RRT utilization, and facilitate quality improvement efforts across diverse settings. The Medical Emergency Team subsection (GWTG-R-MET) within the GWTG-R registry allows participating hospitals to submit medical history, hospital care, and outcomes of patients experiencing RRT activations through an online reporting form. A comprehensive analysis of data from 360 hospitals (2005-2015) was undertaken by Lyons et al. (2019) (LYONS) [9] and offered valuable, large-scale perspectives on RRT characteristics and outcomes. However, despite registry data and widespread RRT implementation, their effectiveness in reducing mortality, cardiopulmonary arrest, unplanned ICU admission, and length of stay (LOS) is still unclear and heterogeneous.

For example, Chan et al. [10] observed a 33.8% reduction in cardiopulmonary arrests without mortality improvements, while Al-Omari et al. [11] reported significant mortality declines. A systematic review by Zhang et al. [12] confirmed this inconsistency, attributing it to heterogeneous settings and protocols. Critically, mid-sized urban community teaching institutions with unique resource constraints and patient populations are underrepresented in this literature. Segon et al. [13] found no mortality reductions in a community-based teaching hospital, highlighting the need for setting specific data.

This study addresses a critical gap in the literature by evaluating the representativeness of national registry data for mid-sized urban community teaching hospitals, settings with distinct patient demographics, acuity levels, and resource constraints that differ from academic or tertiary care centers [14-17]. By comparing local RRT activations to the LYONS study [9], we aim to assess the applicability of national benchmarks to this unique hospital type, identify site-specific factors influencing RRT outcomes, such as trigger patterns and disposition pathways, and highlight the need for local needs assessments.

The primary objective of this study was to compare RRT activation patterns, patient demographics, and clinical outcomes at a mid-sized urban community teaching hospital with national registry data to determine the utility of national benchmarks in informing local quality improvement strategies.

## Materials and methods

This study compares rapid response (RR) events at a mid-sized urban community teaching hospital in the Midwest with data from a multicenter registry analyzed by Lyons et al. [9]. The study period spanned 20 months from July 1, 2022, to March 1, 2024 (n = 418 days), during which the hospital recorded 17,525 unique inpatient admissions. This analysis focused specifically on RR events occurring between 0700 and 1900 hours within this timeframe. The study was reviewed by the Ascension Health Institutional Review Board, and it was determined that the proposed activity is not research involving human subjects as defined by the U.S. Department of Health and Human Services (DHHS)/FDA regulations.

RR events were documented using RR recording sheets and integrated with patient data from the hospital’s Data Warehouse. The Data Warehouse provided information on patient demographics, outcomes, readmission status, and final diagnoses. Final diagnoses were identified using the International Classification of Diseases, Tenth Edition (ICD-10) codes, organized by chapter, sub-chapter, and specific diagnosis code. All data were de-identified by the principal investigator before analysis or sharing.

Raw data on RR events, interventions, and patient dispositions were recoded into representative categories for analysis. Illness categories were derived from final diagnosis and surgical records and simplified into five broad categories. To ensure a valid comparison with the GWTG-R-MET (Get With The Guidelines-Resuscitation - Medical Emergency Team) registry as analyzed by Lyons et al., local data fields were mapped to the registry’s standardized definitions. Specifically, ICD-10 diagnostic codes from the electronic health record were grouped into the five primary GWTG-R illness categories (Medical, Surgical, Obstetric, Trauma, and Other). For patient disposition, local "acuity-adaptable unit" stays were recoded as "Remained on Unit" to match the registry’s binary distinction between ward-level care and escalation to higher levels of care (ICU/Stepdown).

Comparisons were made between the mid-sized urban community teaching hospital and the analyzed findings from the LYONS study [9], which utilized the GWTG-R-MET registry. Raw data from the LYONS study were not directly included; instead, the studied hospital’s data were simplified to align with LYONS’ categories, including demographics, RR triggers, disposition, and mortality. Due to incomplete data from LYONS, formal statistical comparisons for age and length of stay based on median and interquartile ranges were not attempted.

The "Other/Unknown" illness category (26.5%) at the study hospital primarily comprised patients whose primary ICD-10 codes did not clearly align with the four specific GWTG-R categories (Medical, Surgical, Obstetric, Trauma). This included a high volume of patients admitted for observation with vague constitutional symptoms, psychiatric primary diagnoses, or those whose documentation was undergoing final coding at the time of data extraction. In contrast, the LYONS registry benefits from a more mature, standardized data entry interface that may force clinicians to select one of the four primary categories, potentially explaining the lower "Other" rate in the national cohort.

Statistical significance was determined using chi-squared tests, with a p-value of less than 0.05 considered significant. Proportions and key parameters were compared between the studied hospital and LYONS’ data [9], as detailed in the results section. Regarding data integrity, a complete-case analysis approach was utilized. Events with missing primary outcome data or incomplete demographic records (n < 5% of total activations) were excluded from the final comparative analysis to maintain the denominator’s accuracy. For RRT triggers, if multiple triggers were checked for a single event, each was counted as a distinct activation reason, consistent with the registry's reporting structure.

## Results

Data were collected over a 20-month period (July 1, 2022, to March 1, 2024) at a mid-sized urban community teaching hospital. A mean of 2.18 responses occurred per day (SD = 1.45, range = 0-14). During this time, 912 RR events occurred among 783 unique patients, with multiple responses recorded for 103 patients. Significant differences were observed in patient demographics, RRT triggers, and outcomes compared to the national registry data presented by LYONS [9]. Key findings are presented below, including patient characteristics, RRT triggers, dispositions following RRT activation, and the relationship between multiple responses and mortality.

Patient characteristics are summarized in Table 1. The median age of patients at the study hospital was 68 years (IQR: 58-79), compared to 66 years (IQR: 53-78) in the LYONS study [9]. While no significant differences were observed in sex distribution, the study hospital had a significantly higher proportion of non-Hispanic Black patients (75.9% vs. 19.7%, χ2 = 1806.0, p < 0.001) and a lower proportion of non-Hispanic White patients (17.8% vs. 67.2%, χ2 = 1004.0, p < 0.001). Lastly, patients at the study hospital had a similar median length of stay (nine days vs. eight days), although formal statistical testing was not performed. Significant differences were also noted in illness categories, with the study hospital having lower proportions of patients in the medical and surgical categories, and more other/unknown category patients compared to LYONS data (Table 1).

**Table 1:** Patient characteristics.

	LYONS [9]	Study hospital	χ2	p
Patients, n	347,401	912		
Age, median (IQR)	66 (53-78)	68 (58-79)		
Sex, n (%)				
Female	184,613 (53.1)	493 (54.1)	0.271	0.602
Male	162,688 (46.8)	419 (45.9)	0.253	0.615
Other	100 (0.03)			
Race, n (%)				
Non-Hispanic Black	68,307 (19.7)	692 (75.9)	1806.0	<0.001
Non-Hispanic White	233,434 (67.2)	162 (17.8)	1004.0	<0.001
Unknown/not recorded	23,878 (6.9)	49 (5.4)	2.9	0.085
Asian/Pacific Islander	4,471 (1.3)	4 (0.4)	4.5	0.034
Hispanic White	11,367 (3.3)	N/A		
Hispanic Other	5,944 (1.7)	N/A		
Length of stay, median (IQR)	8 (4-15)	9 (5-16)		
Illness category, n (%)				
Medical	272,575 (78.5)	523 (57.3)	238.3	<0.001
Surgical	58,190 (16.8)	125 (13.7)	5.8	0.016
Obstetric	3,078 (0.9)	3 (0.3)	2.6	0.106
Trauma	3,151 (0.9)	19 (2.1)	12.7	<0.001
Other/unknown	10,407 (3.0)	242 (26.5)	1692.6	<0.001

RRT triggers are summarized in Table 2. Neurological triggers were significantly more common at the study hospital compared to LYONS [9] (41.7% vs. 30.7%, χ2 = 51.3, p < 0.001), with higher rates of mental status changes (23.9% vs. 20.5%, χ2 = 6.1, p = 0.014) and suspected acute stroke (6.3% vs. 2.7%, χ2 = 41.8, p < 0.001). Although overall cardiac trigger rates were similar (38.0% vs. 37.4%, χ2 = 0.1, p = 0.725), chest pain was 3.5 times more frequent at the study hospital (16.1% vs. 4.5%, χ2 = 270.0, p < 0.001). Additionally, respiratory triggers were less common at the study hospital (12.7% vs. 38.0%, χ2 = 246.2, p < 0.001), including lower rates of decreased oxygen saturation (5.4% vs. 21.7%, χ2 = 142.7, p < 0.001), new onset of difficulty breathing (5.0% vs. 15.7%, χ2 = 77.8, p < 0.001), and tachypnea (1.9% vs. 12.2%, χ2 = 93.1, p < 0.001). Lastly, LYONS reported more "Other, not specified" RRT activations.

**Table 2:** Rapid response team triggers.

Triggers, n (%)	LYONS [9]	Study hospital	χ2	p
Neurologic	103,735 (30.7)	380 (41.7)	51.3	<0.001
Mental status change	69,532 (20.5)	218 (23.9)	6.1	0.014
Suspected acute stroke	9,166 (2.7)	57 (6.3)	41.8	<0.001
Seizure	15,019 (4.4)	49 (5.4)	1.7	0.197
Acute loss of consciousness	22,143 (6.5)	48 (5.3)	2.2	0.135
Unexplained agitation/delirium	3,239 (1.0)	8 (0.9)	0.0060	0.938
Cardiac	126,654(37.4)	347 (38.0)	0.1	0.725
Chest pain	15,585 (4.6)	147 (16.1)	270.0	<0.001
Hypotension	53,103 (15.7)	112 (12.3)	7.8	0.005
Tachycardia	59,871 (17.7)	81 (8.9)	47.9	<0.001
Bradycardia	13,241 (3.9)	7 (0.77)	23.2	<0.001
Respiratory	128,656 (38.0)	116 (12.7)	246.2	<0.001
Decreased oxygen saturation	73,627 (21.7)	49 (5.4)	142.7	<0.001
New onset of difficulty breathing	53,277 (15.7)	46 (5.0)	77.8	<0.001
Tachypnea	42,237 (12.5)	17 (1.9)	93.1	<0.001
Respiratory depression	17,483 (5.2)	4 (0.44)	40.6	<0.001
Other	149,683 (44.2)	69 (7.6)	494.5	<0.001
Other, not specified	78,598 (22.2)	50 (5.5)	159.8	<0.001
Uncontrolled bleeding	5,076 (1.5)	19 (2.1)	1.7	0.190
Acute decrease in urinary output	1,313 (0.4)	N/A		
Staff member acutely worried about the patient	85,459 (25.3)	N/A		

Disposition patterns following RRT activation are summarized in Table 3. While the majority of patients in both groups remained in the unit where the RRT was activated, this proportion was significantly higher at the study hospital compared to LYONS (70.4% vs. 54.9%, χ2 = 88.0, p < 0.001). Conversely, transfers to the ICU were significantly less frequent at the study hospital (21.5% vs. 30.0%, χ2 = 27.9, p < 0.001).

**Table 3:** Disposition following rapid response team activation.

Disposition, n (%)	LYONS [9]	Study hospital	χ2	p
Remained in the unit	190,627 (54.9)	642 (70.4)	88.0	<0.001
Transfer to ICU	102,609 (30.0)	196 (21.5)	27.9	<0.001
Transfer to telemetry/stepdown	26,209 (7.6)	55 (6.0)	2.7	0.100
Other	24,417 (6.9)	18 (2.0)	34.8	<0.001
Cath laboratory	1,371(0.4)	1 (0.1)	1.3	0.246
Operating room	1,426 (0.4)	N/A		
Transferred to another hospital	742 (0.2)	N/A		

**Figure 1:**
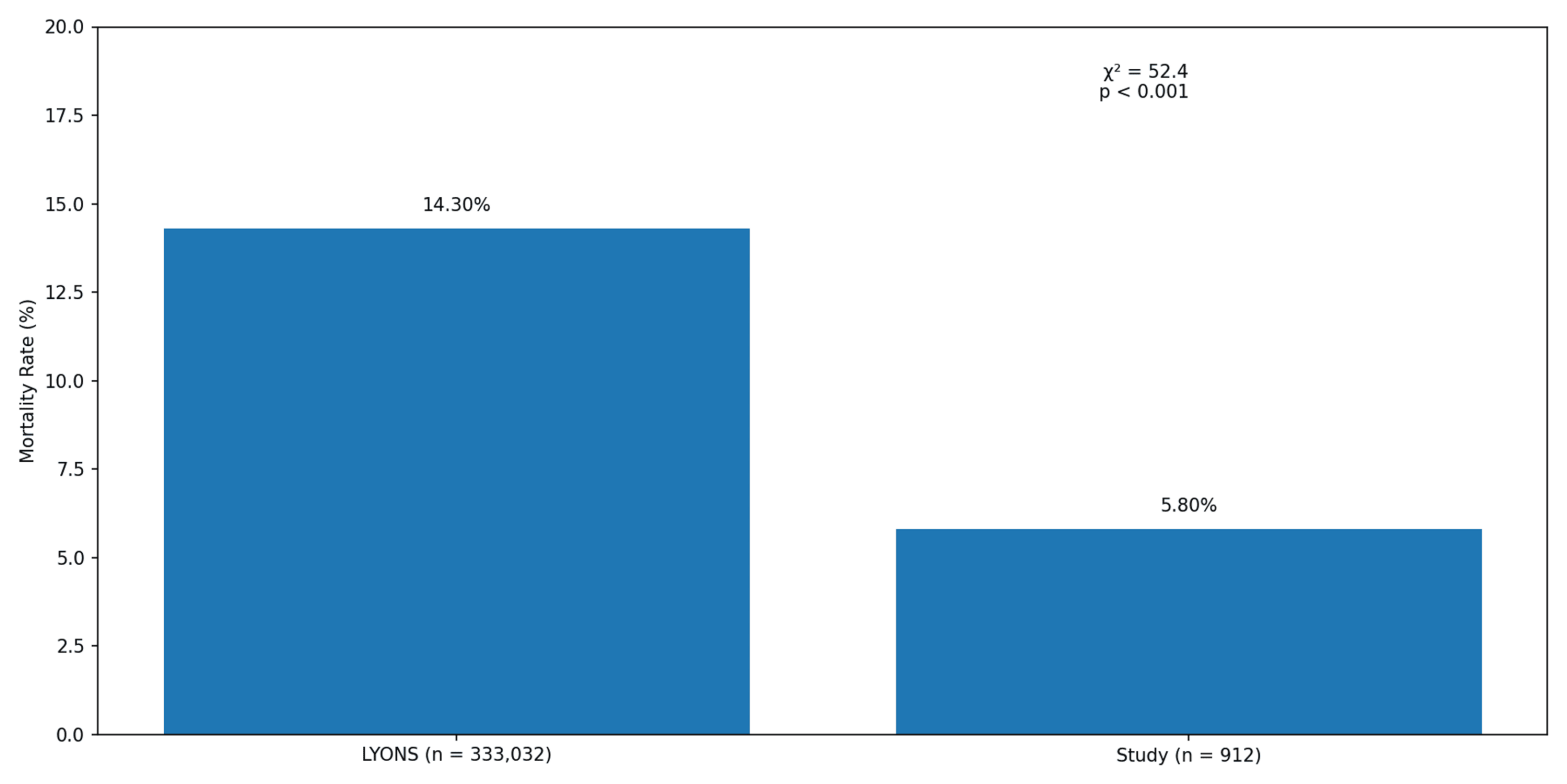
In-hospital mortality rate of patients experiencing rapid response team activations.

In-patient mortality outcomes following RRT activation were compared between the two cohorts (Figure 1). A significantly lower mortality rate was observed at the study hospital (5.8%) compared to LYONS (14.3%, χ2 = 52.4, p < 0.001).

## Discussion

This study compared RRT activations at a mid-sized urban community teaching hospital to national data from the GWTG-R-MET registry to evaluate its representativeness for this hospital type [9]. Significant differences were found in patient demographics, activation triggers, disposition outcomes, and mortality rates, suggesting that national registries may inadequately capture the unique needs and challenges of specific hospital settings. For instance, the study hospital reported higher rates of neurological triggers and chest pain, lower rates of respiratory triggers, a greater proportion of non-Hispanic Black patients, fewer transfers to different units, and lower inpatient mortality following RRT activation. While national registries provide valuable opportunities to develop benchmarks, our results highlight the importance of local needs assessments to ensure institutionally relevant policies and interventions.

Higher rates of neurological triggers, including altered mental status and suspected acute stroke, were observed at the studied hospital. This may reflect a higher prevalence of cardiovascular comorbidities, variation in staff training, or differences in RRT activation criteria. While prior studies note institutional variability in RRT activation policies, such variability is unlikely to fully explain these findings [8,18,19]. The hospital’s designation as a level 1 stroke center may attract patients with a higher risk for cardiovascular disease, potentially contributing to higher neurological trigger rates [20]. Lastly, informal handoffs or inconsistent documentation of neurological status, an issue reported across various hospital settings, could increase RRT activations [21-23]. However, evidence linking documentation gaps to activation rates remains scarce.

The RRT activation rate for chest pain at the studied hospital was 3.5 times higher than in the comparison group, while respiratory triggers were markedly lower. This may reflect the hospital’s cardiac capabilities, including percutaneous coronary intervention (PCI), cardiac surgery, and a dedicated cardiac ICU capable of mechanical circulatory support, which likely attracts a higher acuity patient population with elevated cardiovascular disease risk. Importantly, the data does not differentiate between cardiac and non-cardiac causes of chest pain, and the hospital’s capabilities may bias frontline staff toward cardiac causes. Furthermore, the need for RRT activations for respiratory issues may be reduced by nursing ability to contact respiratory therapists to administer respiratory interventions, and the presence of acuity-adaptable units. Likewise, fewer transfers to other units occurred at our hospital, including ICU transfers. This may be reflective of the hospital’s multiple acuity-adaptable units with staff trained to manage higher acuity patients, stepdown units capable of managing ventilated patients, and multiple floors with telemetry capabilities. However, direct cross-institutional comparisons of acuity-adaptable units, nursing autonomy, hospital capability, and RRT activation rates are absent in the existing literature.

At the studied hospital, non-Hispanic Black patients comprised a significantly larger proportion of RRT activations with higher activation rates. While this may align with community demographics, Lykins et al. found that Black patients are more likely to trigger RRT activations within the first four hours of admission, with subsequent worse outcomes [24]. Additionally, the study hospital reported similar median LOS for patients necessitating RRT activations, and while LOS was not statistically analyzed, these data align with prior studies linking prolonged LOS to higher medical complexity and adverse outcomes [25,26].

Furthermore, a significant difference was observed in inpatient mortality following RRT activation. The study hospital recorded a markedly lower mortality rate. While encouraging, interpreting this finding requires caution. Potential contributing factors include patient acuity at the time of activation (despite similarities in trigger profiles), effectiveness of local RRT interventions and post-RRT care pathways, impact of acuity-adaptable units allowing earlier intervention or management without formal ICU transfer, variations in reporting/coding practices between institutions, or the influence of the different time periods analyzed. The lower rate could reflect successful RRT interventions preventing progression to irreversible states, though the specific reasons warrant further investigation. This difference in a critical outcome further emphasizes that direct application of national mortality benchmarks without considering local context and potential mediating factors may be misleading.

Our findings are consistent with prior studies highlighting institutional variability in RRT outcomes, but further this understanding by demonstrating how mid-sized urban community teaching hospitals differ from national registries [12]. For instance, the higher proportion of non-Hispanic Black patients in our cohort mirrors disparities observed by Lykins et al., who identified earlier RRT activations and worse outcomes in Black patients [24]. This may suggest inequities in care access within local communities that may disproportionately affect the non-Hispanic Black population. Similarly, the elevated neurological trigger rates and lower mortality at the studied hospital contrast with LYONS’ data [9], further suggesting institutional heterogeneity in trigger patterns and outcomes. These differences underscore the limitations of generalizing registry data to individual hospitals.

These findings have important implications as they emphasize the importance of local needs assessments to identify high-frequency triggers and tailor RRT training and resource allocation accordingly. For example, at the studied institution, training of frontline staff to evaluate and differentiate cardiac and non-cardiac causes of chest pain may reduce RRT activations, and improvement in documentation and handoff policies may reduce activations for neurological triggers. Additionally, while activation criteria may align with other institutions, hospitals should develop protocols for high-frequency local triggers to reduce variability in care. Furthermore, the overrepresentation of non-Hispanic Black patients in RRT activations suggests that targeted interventions, such as implicit bias training and community outreach, may address health disparities. Lastly, acuity-adaptable units and nursing autonomy in respiratory care may explain the lower transfer and respiratory trigger rates at the studied hospital, suggesting that empowering frontline staff could reduce avoidable escalations.

This study provides critical insight into RRT activations in mid-sized urban community teaching hospitals; however, its findings must be interpreted in the context of its strengths and limitations. Notable strengths include its focus on an underrepresented setting, actionable local insights, and methodological rigor. The studied hospital type is underrepresented in national registries and often differs markedly in patient demographics, resource availability, and staffing models. These differences highlight the unique challenges faced by community hospitals serving socioeconomically diverse populations. Additionally, it provides actionable data for similar hospitals; higher rates of neurological and chest pain triggers underscore the need for targeted staff training, particularly in institutions that specialize in stroke and cardiac care. Lastly, despite a smaller sample size (n = 912) compared to LYONS (n = 347,401), the study’s 20-month data collection period ensures relevance to current practice [9].

However, in light of these strengths, limitations to consider include the comparison across different time periods, exclusion of nighttime activations, single-center design, sample size, differences in data granularity between the registry and local data (for example, LYONS reported a higher proportion of triggers as "Other, not specified," limiting direct comparison for this category), and limited longitudinal outcomes. The LYONS registry data span 2005-2015, while our data are from 2022 to 2024; national RRT practices, patient populations, and general hospital care may have evolved during this interval, potentially influencing comparisons. The studied data were collected between 0700 and 1900, excluding nighttime activations. Critically, nighttime shifts often have reduced staffing, with some studies suggesting higher mortality rates [27,28]. Excluding high-risk periods (0600-0800 and 2300-2400) may bias the data toward daytime outcomes. While the 5.8% vs. 14.3% gap is wide, a portion of this variance is a byproduct of this selection bias rather than a pure reflection of superior clinical outcomes. However, this requires further investigation, as it is unclear if this is due to timing of shift changes, nighttime staffing structures, influence of circadian rhythm, or other unstudied causes [29,30]. Additionally, the single-center design limits generalizability to other community hospitals, particularly non-teaching or rural institutions. While the sample size (n = 912) is reasonable, it lacks the statistical power of multi-center studies for detecting smaller differences. Review of our results must consider the specific clinical infrastructure of the study hospital. As a level 1 stroke center with comprehensive cardiac surgical and mechanical circulatory support capabilities, our institution represents a high-resource tier of the "community teaching hospital" category. The markedly higher rates of neurological and chest pain triggers likely reflect this specific service tilt. Consequently, our findings may not be directly generalizable to smaller, non-teaching, or rural community hospitals that lack such specialized service lines. Lastly, the study focuses on in-hospital outcomes but does not track post-discharge outcomes such as readmission rates or long-term survival. This limits the understanding of the overall effectiveness of RRT on patient care.

Future research should explore the relationship between informal handoffs or inconsistent documentation and RRT activations to identify vital areas of quality improvement. Additionally, future research should include multi-center studies to compare RRT outcomes across similar hospital settings and time periods to identify best practice policies. Also, national registries could enhance data collection to include operational data like staffing ratios throughout a 24-hour period, hospital capabilities (e.g., acuity-adaptable unit prevalence), more granular trigger categories, and community health indices to improve cross-institutional comparisons and understanding of higher nighttime mortality. Lastly, investigation of potential disparities in long-term outcomes for non-Hispanic Black patients experiencing higher rates of RRT activations may inform targeted policies to improve patient care.

## Conclusions

In conclusion, this study highlights significant differences in RRT activations, outcomes, and patient characteristics at a mid-sized urban community teaching hospital compared to national registry data. By revealing unique patient demographics, trigger patterns, disposition pathways, and mortality rates, our findings emphasize the need to supplement national benchmarks with local data to identify and address site-specific contributors to RRT patterns. By understanding and addressing these unique institution-specific challenges, hospitals can improve the effectiveness of their RR systems and enhance patient outcomes by ensuring interventions are tailored to the hospital’s unique patient population and operational context.

## References

[1] McQuillan P, Pilkington S, Allan A (1998). Confidential inquiry into quality of care before admission to intensive care. BMJ.

[2] Naik H, Murray TM, Khan M (2024). Population-based trends in complexity of hospital inpatients. JAMA Intern Med.

[3] Devita MA, Bellomo R, Hillman K (2006). Findings of the first consensus conference on medical emergency teams. Crit Care Med.

[4] Hodgetts TJ, Kenward G, Vlackonikolis I (2002). Incidence, location and reasons for avoidable in-hospital cardiac arrest in a district general hospital. Resuscitation.

[5] Schein RM, Hazday N, Pena M, Ruben BH, Sprung CL (1990). Clinical antecedents to in-hospital cardiopulmonary arrest. Chest.

[6] Smith AF, Wood J (1998). Can some in-hospital cardio-respiratory arrests be prevented? A prospective survey. Resuscitation.

[7] Stolldorf DP, Jones CB (2015). Deployment of rapid response teams by 31 hospitals in a statewide collaborative. Jt Comm J Qual Patient Saf.

[8] Mitchell OJ, Motschwiller CW, Horowitz JM, Evans LE, Mukherjee V (2019). Characterising variation in composition and activation criteria of rapid response and cardiac arrest teams: a survey of Medicare participating hospitals in five American states. BMJ Open.

[9] Lyons PG, Edelson DP, Carey KA (2019). Characteristics of rapid response calls in the United States: an analysis of the first 402,023 adult cases from the Get With The Guidelines Resuscitation-Medical Emergency Team Registry. Crit Care Med.

[10] Chan PS, Jain R, Nallmothu BK, Berg RA, Sasson C (2010). Rapid response teams: a systematic review and meta-analysis. Arch Intern Med.

[11] Al-Omari A, Al Mutair A, Aljamaan F (2019). Outcomes of rapid response team implementation in tertiary private hospitals: a prospective cohort study. Int J Emerg Med.

[12] Zhang Q, Lee K, Mansor Z, Ismail I, Guo Y, Xiao Q, Lim PY (2024). Effects of a rapid response team on patient outcomes: a systematic review. Heart Lung.

[13] Segon A, Ahmad S, Segon Y, Kumar V, Friedman H, Ali M (2014). Effect of a rapid response team on patient outcomes in a community-based teaching hospital. J Grad Med Educ.

[14] Caveney AF, Silbergleit R, Frederiksen S, Meurer WJ, Hickenbottom SL, Smith RW, Scott PA (2010). Resource utilization and outcome at a university versus a community teaching hospital in tPA treated stroke patients: a retrospective cohort study. BMC Health Serv Res.

[15] Colquhoun C, Hafeez MR, Heath K, Hays R (2009). Aligning clinical resources to curriculum needs: the utility of a group of teaching hospitals. Med Teach.

[16] Kuwabara K, Matsuda S, Fushimi K, Ishikawa KB, Horiguchi H, Hayashida K, Fujimori K (2011). Contribution of case-mix classification to profiling hospital characteristics and productivity. Int J Health Plann Manage.

[17] Williams JL, Lugg D, Gray R, Hollis D, Stoner M, Stevenson R (2010). Patient demographics, complications, and hospital utilization in 250 consecutive device implants in a new community hospital electrophysiology program--implications for 'niche' hospitals. Am Heart Hosp J.

[18] Kim SW, Lee HY, Han MR (2017). Epidemiology and clinical characteristics of rapid response team activations. Korean J Crit Care Med.

[19] Reardon PM, Fernando SM, Murphy K, Rosenberg E, Kyeremanteng K (2018). Factors associated with delayed rapid response team activation. J Crit Care.

[20] Chaudhry SA, Afzal MR, Chaudhry BZ (2016). Rates of adverse events and outcomes among stroke patients admitted to primary stroke centers. J Stroke Cerebrovasc Dis.

[21] Banzon PC, Vashisht A, Euckert M, Nairon E, Aiyagari V, Stutzman SE, Olson DM (2023). Original research: Practice variations in documenting neurologic examinations in non-neuroscience ICUs. Am J Nurs.

[22] Sarko J (2009). Emergency medicine residents do not document detailed neurologic examinations. Acad Emerg Med.

[23] Tucci V, Laufman L, Peacock WF, Moukaddam N, Shah A, Peacock WF (2016). Epic fail! Poor neuropsychiatric documentation practices in emergency psychiatric patients. Emerg Med.

[24] Lykins V JD, Freedman MT, Zemore Z (2023). Patients who decompensate and trigger rapid response immediately upon hospital admission have higher mortality than equivalent patients without rapid responses. J Patient Saf.

[25] Ameri M, Ali N, Dickson K, Koom-Dabzie K, Al-Ameri A (2022). Analysis of extended length of stay in a comprehensive cancer center. Recent Adv Clin Trials.

[26] Anis HK, Sodhi N, Acuña AJ (2021). Does increasing patient complexity have an effect on medical outcomes and lengths-of-stay after total knee arthroplasty?. J Knee Surg.

[27] Churpek MM, Edelson DP, Lee JY, Carey K, Snyder A (2017). Association between survival and time of day for rapid response team calls in a national registry. Crit Care Med.

[28] Fernando SM, Reardon PM, Bagshaw SM (2018). Impact of nighttime rapid response team activation on outcomes of hospitalized patients with acute deterioration. Crit Care.

[29] Boniatti MM, de Loreto MS, Mazzutti G (2023). Association between time of day for rapid response team activation and mortality. J Crit Care.

[30] Vicent L, González-Casal D, Bruña V (2020). Circadian rhythm of deaths in a cardiology department: a five-year analysis. Cardiology.

